# Supplementary material for: Single Cell Transcriptomic and Chromatin Profiles Suggest Layer Vb Is the Only Layer With Shared Excitatory Cell Types in the Medial and Lateral Entorhinal Cortex
Source: Front Neural Circuits. 2022 Jan 26;15:806154. doi: 10.3389/fncir.2021.806154 (PMC8826650; doi:10.3389/fncir.2021.806154)
Supplement: Supplementary file 14 [file Data_Sheet_1.ZIP › Deep_vs_Sup_Motifs/knownResults.html]

Deep\_vs\_Sup\_Motifs/ - Homer Known Motif Enrichment Results


# Homer Known Motif Enrichment Results (Deep\_vs\_Sup\_Motifs/)

Homer *de novo* Motif Results  
Gene Ontology Enrichment Results  
Known Motif Enrichment Results (txt file)  
Total Target Sequences = 9298, Total Background Sequences = 32571

|  |  |  |  |  |  |  |  |  |  |  |  |
| --- | --- | --- | --- | --- | --- | --- | --- | --- | --- | --- | --- |
| Rank | Motif | Name | P-value | log P-pvalue | q-value (Benjamini) | # Target Sequences with Motif | % of Targets Sequences with Motif | # Background Sequences with Motif | % of Background Sequences with Motif | Motif File | SVG |
| 1 | A G C T A C G T A C T G A T G C A G T C C G T A C T G A T A C G | NF1-halfsite(CTF)/LNCaP-NF1-ChIP-Seq(Unpublished)/Homer | 1e-127 | -2.931e+02 | 0.0000 | 4927.0 | 52.99% | 13227.2 | 40.61% | motif file (matrix) | svg |
| 2 | A G C T A G T C A T G C A G C T A C G T C G T A A C G T A G T C C G A T A T G C | Gata2(Zf)/K562-GATA2-ChIP-Seq(GSE18829)/Homer | 1e-83 | -1.930e+02 | 0.0000 | 2326.0 | 25.02% | 5544.3 | 17.02% | motif file (matrix) | svg |
| 3 | T A C G A T G C G C T A A C T G C G T A A C G T C G T A C T G A T A C G T C G A | Gata4(Zf)/Heart-Gata4-ChIP-Seq(GSE35151)/Homer | 1e-83 | -1.921e+02 | 0.0000 | 3373.0 | 36.28% | 8813.9 | 27.06% | motif file (matrix) | svg |
| 4 | T A G C G C T A A C T G C G T A A C G T C G T A C G T A T A C G T C A G T C G A | Gata1(Zf)/K562-GATA1-ChIP-Seq(GSE18829)/Homer | 1e-82 | -1.900e+02 | 0.0000 | 2042.0 | 21.96% | 4716.6 | 14.48% | motif file (matrix) | svg |
| 5 | A G C T A T G C G A C T G C A T C G T A A G C T G T A C C G A T A T C G A G T C | Gata6(Zf)/HUG1N-GATA6-ChIP-Seq(GSE51936)/Homer | 1e-78 | -1.800e+02 | 0.0000 | 3165.0 | 34.04% | 8238.5 | 25.29% | motif file (matrix) | svg |
| 6 | G C T A A T C G G C T A G A C T G C T A T C G A T A G C T C G A | GATA3(Zf)/iTreg-Gata3-ChIP-Seq(GSE20898)/Homer | 1e-75 | -1.738e+02 | 0.0000 | 4668.0 | 50.20% | 13263.7 | 40.72% | motif file (matrix) | svg |
| 7 | C G T A A C T G C G T A A C G T C G T A C T G A T C A G T G C A A G C T T G A C | TRPS1(Zf)/MCF7-TRPS1-ChIP-Seq(GSE107013)/Homer | 1e-71 | -1.640e+02 | 0.0000 | 5826.0 | 62.66% | 17413.0 | 53.46% | motif file (matrix) | svg |
| 8 | C T G A C T G A C A T G A T C G A G C T A T C G G A C T C A T G C T G A G T C A | Tbr1(T-box)/Cortex-Tbr1-ChIP-Seq(GSE71384)/Homer | 1e-54 | -1.248e+02 | 0.0000 | 4641.0 | 49.91% | 13645.6 | 41.89% | motif file (matrix) | svg |
| 9 | C T G A C T A G A C T G G C A T C T A G G C A T A T C G C T G A C G T A G T C A | Tbx21(T-box)/GM12878-TBX21-ChIP-Seq(Encode)/Homer | 1e-42 | -9.803e+01 | 0.0000 | 3498.0 | 37.62% | 10068.9 | 30.91% | motif file (matrix) | svg |
| 10 | G C T A G A C T G A C T T G C A C G T A A G T C C G T A T A G C G A T C G A C T | Eomes(T-box)/H9-Eomes-ChIP-Seq(GSE26097)/Homer | 1e-37 | -8.701e+01 | 0.0000 | 5970.0 | 64.21% | 18772.1 | 57.63% | motif file (matrix) | svg |
| 11 | C G T A C T A G C A T G A G C T A C T G C G A T A T C G C G T A G T C A G T C A | Tbet(T-box)/CD8-Tbet-ChIP-Seq(GSE33802)/Homer | 1e-34 | -8.040e+01 | 0.0000 | 3573.0 | 38.43% | 10526.3 | 32.32% | motif file (matrix) | svg |
| 12 | A C G T C T A G C G T A A G T C G T A C A C G T A C G T A C G T G T C A G T A C T G A C G A C T | Nur77(NR)/K562-NR4A1-ChIP-Seq(GSE31363)/Homer | 1e-33 | -7.615e+01 | 0.0000 | 751.0 | 8.08% | 1661.7 | 5.10% | motif file (matrix) | svg |
| 13 | A G C T C T A G T G A C C G T A A C G T C G A T A G T C A G T C C T G A C A T G | TEAD3(TEA)/HepG2-TEAD3-ChIP-Seq(Encode)/Homer | 1e-29 | -6.848e+01 | 0.0000 | 3505.0 | 37.70% | 10452.7 | 32.09% | motif file (matrix) | svg |
| 14 | G A C T C G A T T C A G G A T C G A C T A G C T A G C T A G T C G A T C C G T A C T A G C T A G T C G A T C G A C T G A | Bcl6(Zf)/Liver-Bcl6-ChIP-Seq(GSE31578)/Homer | 1e-27 | -6.407e+01 | 0.0000 | 4072.0 | 43.79% | 12439.9 | 38.19% | motif file (matrix) | svg |
| 15 | C T A G C A G T C G T A A C G T A G T C A C T G C G T A A G C T A G T C G A T C | HNF6(Homeobox)/Liver-Hnf6-ChIP-Seq(ERP000394)/Homer | 1e-25 | -5.907e+01 | 0.0000 | 1681.0 | 18.08% | 4600.3 | 14.12% | motif file (matrix) | svg |
| 16 | G C T A A C T G T C G A C T G A C T G A A C G T T A G C C T G A C G T A C G A T | Cux2(Homeobox)/Liver-Cux2-ChIP-Seq(GSE35985)/Homer | 1e-25 | -5.850e+01 | 0.0000 | 1450.0 | 15.59% | 3881.8 | 11.92% | motif file (matrix) | svg |
| 17 | A T C G G A T C G C A T C G T A G T C A A C G T A T G C A G T C | CRX(Homeobox)/Retina-Crx-ChIP-Seq(GSE20012)/Homer | 1e-25 | -5.813e+01 | 0.0000 | 5786.0 | 62.23% | 18525.0 | 56.88% | motif file (matrix) | svg |
| 18 | T G A C A G T C C T G A T G A C C G T A A C G T A C G T A G T C A G T C C G T A | TEAD1(TEAD)/HepG2-TEAD1-ChIP-Seq(Encode)/Homer | 1e-23 | -5.474e+01 | 0.0000 | 2956.0 | 31.79% | 8805.1 | 27.03% | motif file (matrix) | svg |
| 19 | T C G A T A G C G T C A A C T G A C T G C G T A C G T A C T A G A G C T T C A G | ERG(ETS)/VCaP-ERG-ChIP-Seq(GSE14097)/Homer | 1e-23 | -5.426e+01 | 0.0000 | 3914.0 | 42.10% | 12048.0 | 36.99% | motif file (matrix) | svg |
| 20 | A G T C G A C T C A G T A C T G C T A G T G A C G C T A A T G C G C A T A T C G C G A T A C T G G A T C G T A C G T C A C T G A | NF1(CTF)/LNCAP-NF1-ChIP-Seq(Unpublished)/Homer | 1e-23 | -5.425e+01 | 0.0000 | 1099.0 | 11.82% | 2840.0 | 8.72% | motif file (matrix) | svg |
| 21 | A T G C T C G A A G T C A G C T A C G T G T A C A G T C G C T A C T A G C A T G G T C A C T G A T C A G A G T C | Stat3+il21(Stat)/CD4-Stat3-ChIP-Seq(GSE19198)/Homer | 1e-20 | -4.732e+01 | 0.0000 | 2207.0 | 23.74% | 6436.4 | 19.76% | motif file (matrix) | svg |
| 22 | G A C T C T A G A T G C A G T C G T C A T A C G A T G C A T C G | HIC1(Zf)/Treg-ZBTB29-ChIP-Seq(GSE99889)/Homer | 1e-20 | -4.671e+01 | 0.0000 | 4296.0 | 46.20% | 13488.0 | 41.41% | motif file (matrix) | svg |
| 23 | T A G C T A G C G C A T C A T G A C T G G C T A C G T A A C G T A C T G G A T C | TEAD4(TEA)/Tropoblast-Tead4-ChIP-Seq(GSE37350)/Homer | 1e-20 | -4.607e+01 | 0.0000 | 2415.0 | 25.97% | 7137.1 | 21.91% | motif file (matrix) | svg |
| 24 | T C A G A G C T A C G T A C G T G T A C G A T C C G T A C T A G C A T G G T C A C G T A T C G A | STAT4(Stat)/CD4-Stat4-ChIP-Seq(GSE22104)/Homer | 1e-19 | -4.557e+01 | 0.0000 | 3232.0 | 34.76% | 9872.0 | 30.31% | motif file (matrix) | svg |
| 25 | C T G A A C G T A C G T A C G T A G T C G A C T C G A T C T G A A C T G C G T A C G T A T C G A | STAT5(Stat)/mCD4+-Stat5-ChIP-Seq(GSE12346)/Homer | 1e-18 | -4.335e+01 | 0.0000 | 1404.0 | 15.10% | 3899.0 | 11.97% | motif file (matrix) | svg |
| 26 | T C G A C T G A T A G C T G A C T C A G T C A G C G T A C G T A T C A G A G C T | ETV1(ETS)/GIST48-ETV1-ChIP-Seq(GSE22441)/Homer | 1e-18 | -4.240e+01 | 0.0000 | 3192.0 | 34.33% | 9790.2 | 30.06% | motif file (matrix) | svg |
| 27 | G C A T C G T A C G A T G A C T A C T G C T G A G A C T G A T C | Hnf6b(Homeobox)/LNCaP-Hnf6b-ChIP-Seq(GSE106305)/Homer | 1e-17 | -4.063e+01 | 0.0000 | 2634.0 | 28.33% | 7947.8 | 24.40% | motif file (matrix) | svg |
| 28 | T C A G C T A G C T A G T G C A C G A T C G A T C G T A C T A G | GSC(Homeobox)/FrogEmbryos-GSC-ChIP-Seq(DRA000576)/Homer | 1e-17 | -4.053e+01 | 0.0000 | 3524.0 | 37.90% | 10949.6 | 33.62% | motif file (matrix) | svg |
| 29 | A T C G G A C T C G A T C G T A C G T A C A G T G A T C G A T C G A T C A G C T | Otx2(Homeobox)/EpiLC-Otx2-ChIP-Seq(GSE56098)/Homer | 1e-17 | -4.050e+01 | 0.0000 | 2449.0 | 26.34% | 7334.2 | 22.52% | motif file (matrix) | svg |
| 30 | C T G A T A G C T G A C T C A G C T A G G T C A C G T A T C A G A G C T T C A G | ETV4(ETS)/HepG2-ETV4-ChIP-Seq(ENCODE)/Homer | 1e-16 | -3.812e+01 | 0.0000 | 2328.0 | 25.04% | 6971.4 | 21.40% | motif file (matrix) | svg |
| 31 | A G T C C G A T A C T G A T C G T G A C G C T A C A T G A T C G T G A C C G A T A C T G T A G C G T A C G T C A | Tlx?(NR)/NPC-H3K4me1-ChIP-Seq(GSE16256)/Homer | 1e-16 | -3.772e+01 | 0.0000 | 1144.0 | 12.30% | 3144.8 | 9.66% | motif file (matrix) | svg |
| 32 | T C G A T A G C T G C A A C T G A C T G C G T A C G T A C T A G G A C T T A C G | ETS1(ETS)/Jurkat-ETS1-ChIP-Seq(GSE17954)/Homer | 1e-16 | -3.755e+01 | 0.0000 | 2616.0 | 28.14% | 7938.5 | 24.37% | motif file (matrix) | svg |
| 33 | A T G C C T G A G A C T A C G T A C G T G T A C G A T C C G A T C T A G C A T G C G T A C G T A C T G A G A C T | STAT1(Stat)/HelaS3-STAT1-ChIP-Seq(GSE12782)/Homer | 1e-15 | -3.664e+01 | 0.0000 | 1181.0 | 12.70% | 3273.0 | 10.05% | motif file (matrix) | svg |
| 34 | C G A T C T G A C G T A C A G T A G T C G A T C G A T C A C T G | Pitx1(Homeobox)/Chicken-Pitx1-ChIP-Seq(GSE38910)/Homer | 1e-15 | -3.595e+01 | 0.0000 | 7923.0 | 85.21% | 26726.8 | 82.06% | motif file (matrix) | svg |
| 35 | C T A G C T G A T C A G A T C G A G C T C A T G G A C T A G T C C T G A T G C A | Tbx6(T-box)/ESC-Tbx6-ChIP-Seq(GSE93524)/Homer | 1e-14 | -3.432e+01 | 0.0000 | 3134.0 | 33.71% | 9739.8 | 29.90% | motif file (matrix) | svg |
| 36 | A C G T T G C A A G C T G A T C C T A G C T G A A G C T G T C A T C G A C G T A | CUX1(Homeobox)/K562-CUX1-ChIP-Seq(GSE92882)/Homer | 1e-14 | -3.399e+01 | 0.0000 | 2113.0 | 22.73% | 6327.0 | 19.43% | motif file (matrix) | svg |
| 37 | G A T C G T A C C G A T A C T G A C T G C G T A C G T A A C G T A C T G G A T C | TEAD(TEA)/Fibroblast-PU.1-ChIP-Seq(Unpublished)/Homer | 1e-14 | -3.398e+01 | 0.0000 | 2181.0 | 23.46% | 6552.2 | 20.12% | motif file (matrix) | svg |
| 38 | C T G A A T G C C G T A A C G T A G T C A G T C A C G T A C T G A T C G G C A T | SPDEF(ETS)/VCaP-SPDEF-ChIP-Seq(SRA014231)/Homer | 1e-14 | -3.396e+01 | 0.0000 | 2597.0 | 27.93% | 7937.6 | 24.37% | motif file (matrix) | svg |
| 39 | A T G C G A T C C G A T C T A G A C T G G C T A C G T A A G C T A C T G A G C T | TEAD2(TEA)/Py2T-Tead2-ChIP-Seq(GSE55709)/Homer | 1e-14 | -3.307e+01 | 0.0000 | 1577.0 | 16.96% | 4587.5 | 14.08% | motif file (matrix) | svg |
| 40 | A T G C A G T C C T G A A G T C C G A T A C G T A G T C A G T C A C G T A T C G G A C T A C G T | Etv2(ETS)/ES-ER71-ChIP-Seq(GSE59402)/Homer | 1e-14 | -3.263e+01 | 0.0000 | 2440.0 | 26.24% | 7436.5 | 22.83% | motif file (matrix) | svg |
| 41 | T G A C C T A G T C A G G T C A C G T A T C A G C G A T T C A G T C G A T G C A C T G A T A G C | PU.1-IRF(ETS:IRF)/Bcell-PU.1-ChIP-Seq(GSE21512)/Homer | 1e-14 | -3.243e+01 | 0.0000 | 3798.0 | 40.85% | 12043.5 | 36.98% | motif file (matrix) | svg |
| 42 | T A G C T C A G C A T G G C A T A G C T C G A T A T G C C G T A C G T A G T C A | CHR(?)/Hela-CellCycle-Expression/Homer | 1e-13 | -3.220e+01 | 0.0000 | 2549.0 | 27.41% | 7808.9 | 23.97% | motif file (matrix) | svg |
| 43 | A G T C G A C T C A G T G T A C A G T C A T C G T C A G A C T G G T C A C G T A | Stat3(Stat)/mES-Stat3-ChIP-Seq(GSE11431)/Homer | 1e-13 | -3.056e+01 | 0.0000 | 1469.0 | 15.80% | 4274.1 | 13.12% | motif file (matrix) | svg |
| 44 | C T A G C G T A G T C A C G T A A G T C G A T C A G C T C T A G C G T A A C G T G T C A G A T C | Six2(Homeobox)/NephronProgenitor-Six2-ChIP-Seq(GSE39837)/Homer | 1e-13 | -3.013e+01 | 0.0000 | 3023.0 | 32.51% | 9445.8 | 29.00% | motif file (matrix) | svg |
| 45 | A G C T C A T G G C A T G A T C T G C A C T A G G A T C A C G T | Tgif2(Homeobox)/mES-Tgif2-ChIP-Seq(GSE55404)/Homer | 1e-12 | -2.968e+01 | 0.0000 | 7014.0 | 75.44% | 23474.4 | 72.07% | motif file (matrix) | svg |
| 46 | A G T C C T G A A G T C C G A T C A G T G A T C A T G C A C T G A T C G G A C T | Fli1(ETS)/CD8-FLI-ChIP-Seq(GSE20898)/Homer | 1e-12 | -2.951e+01 | 0.0000 | 2511.0 | 27.01% | 7731.5 | 23.74% | motif file (matrix) | svg |
| 47 | C G T A T A G C T A G C T G C A A C T G C T A G C G T A C G T A T C A G G A C T | EHF(ETS)/LoVo-EHF-ChIP-Seq(GSE49402)/Homer | 1e-12 | -2.919e+01 | 0.0000 | 3442.0 | 37.02% | 10891.4 | 33.44% | motif file (matrix) | svg |
| 48 | T C G A A G C T A C G T A C G T A G T C A G T C A C G T A T C G G A C T A T C G | EWS:ERG-fusion(ETS)/CADO\_ES1-EWS:ERG-ChIP-Seq(SRA014231)/Homer | 1e-12 | -2.898e+01 | 0.0000 | 2311.0 | 24.85% | 7072.8 | 21.71% | motif file (matrix) | svg |
| 49 | G A C T C A G T A G C T C G A T A G T C G A T C A G T C C G T A A T G C T C A G | Rbpj1(?)/Panc1-Rbpj1-ChIP-Seq(GSE47459)/Homer | 1e-11 | -2.653e+01 | 0.0000 | 3865.0 | 41.57% | 12403.2 | 38.08% | motif file (matrix) | svg |
| 50 | A G C T G C A T A C T G A C G T A G T C A C G T C T A G T A C G | Smad3(MAD)/NPC-Smad3-ChIP-Seq(GSE36673)/Homer | 1e-11 | -2.583e+01 | 0.0000 | 5882.0 | 63.26% | 19486.6 | 59.83% | motif file (matrix) | svg |
| 51 | C T G A C A G T C T G A A G T C C T A G G A C T A T C G G T A C | HIF-1b(HLH)/T47D-HIF1b-ChIP-Seq(GSE59937)/Homer | 1e-10 | -2.532e+01 | 0.0000 | 2134.0 | 22.95% | 6551.6 | 20.11% | motif file (matrix) | svg |
| 52 | T C G A T C G A T A G C G T A C T C A G T A C G C G T A C G T A T C A G A G C T | GABPA(ETS)/Jurkat-GABPa-ChIP-Seq(GSE17954)/Homer | 1e-9 | -2.288e+01 | 0.0000 | 2023.0 | 21.76% | 6229.2 | 19.12% | motif file (matrix) | svg |
| 53 | C T G A A G T C C G A T A G C T A T G C G T A C A C G T A T C G C A G T G C A T | Elf4(ETS)/BMDM-Elf4-ChIP-Seq(GSE88699)/Homer | 1e-9 | -2.151e+01 | 0.0000 | 2463.0 | 26.49% | 7735.6 | 23.75% | motif file (matrix) | svg |
| 54 | G A C T G C A T C T A G C G A T G A T C T C G A C A T G G A T C | Tgif1(Homeobox)/mES-Tgif1-ChIP-Seq(GSE55404)/Homer | 1e-9 | -2.134e+01 | 0.0000 | 6722.0 | 72.30% | 22606.0 | 69.41% | motif file (matrix) | svg |
| 55 | C T G A A G C T A C G T A C G T A G T C G A C T G A C T C T G A C T G A C T A G C G T A C G T A | STAT6(Stat)/CD4-Stat6-ChIP-Seq(GSE22104)/Homer | 1e-8 | -2.039e+01 | 0.0000 | 2055.0 | 22.10% | 6388.0 | 19.61% | motif file (matrix) | svg |
| 56 | G A C T C A G T G A T C G A T C A C G T G A T C C T G A T A C G C G T A G T C A | STAT6(Stat)/Macrophage-Stat6-ChIP-Seq(GSE38377)/Homer | 1e-8 | -2.012e+01 | 0.0000 | 2064.0 | 22.20% | 6424.5 | 19.72% | motif file (matrix) | svg |
| 57 | G C A T A T C G C A T G G T A C G C T A A G T C T C A G T G A C G T C A T G C A | Arnt:Ahr(bHLH)/MCF7-Arnt-ChIP-Seq(Lo\_et\_al.)/Homer | 1e-8 | -1.970e+01 | 0.0000 | 1312.0 | 14.11% | 3938.5 | 12.09% | motif file (matrix) | svg |
| 58 | T G C A C G T A G T C A A G C T A G T C G C T A T A G C C G A T C T A G G A T C | Gfi1b(Zf)/HPC7-Gfi1b-ChIP-Seq(GSE22178)/Homer | 1e-8 | -1.952e+01 | 0.0000 | 1940.0 | 20.86% | 6022.3 | 18.49% | motif file (matrix) | svg |
| 59 | C G T A T G A C T A G C T G C A A C T G A C T G C G T A C G T A T C A G G A C T | ELF3(ETS)/PDAC-ELF3-ChIP-Seq(GSE64557)/Homer | 1e-8 | -1.951e+01 | 0.0000 | 2133.0 | 22.94% | 6669.2 | 20.48% | motif file (matrix) | svg |
| 60 | C G T A C G A T G A C T G A C T T G A C C T G A A T G C C T G A T A G C A G T C A C G T T C G A C A T G T A C G G A C T A T C G G A C T A C G T C T G A T C G A C G T A | Brachyury(T-box)/Mesoendoderm-Brachyury-ChIP-exo(GSE54963)/Homer | 1e-8 | -1.915e+01 | 0.0000 | 1027.0 | 11.05% | 3019.6 | 9.27% | motif file (matrix) | svg |
| 61 | T G C A C T G A A G T C G T C A A C T G A C T G C G T A C G T A C T G A A G C T | EWS:FLI1-fusion(ETS)/SK\_N\_MC-EWS:FLI1-ChIP-Seq(SRA014231)/Homer | 1e-8 | -1.906e+01 | 0.0000 | 1478.0 | 15.90% | 4496.6 | 13.81% | motif file (matrix) | svg |
| 62 | T C A G A T C G G A C T A C T G G A C T C A G T C T A G C G T A G T A C C G T A C T A G A T C G | Tbx20(T-box)/Heart-Tbx20-ChIP-Seq(GSE29636)/Homer | 1e-7 | -1.832e+01 | 0.0000 | 661.0 | 7.11% | 1860.0 | 5.71% | motif file (matrix) | svg |
| 63 | C G T A C A T G C A T G A C T G C T A G T C G A G C A T C G A T A G C T A G T C G A T C G T A C | NFkB-p65(RHD)/GM12787-p65-ChIP-Seq(GSE19485)/Homer | 1e-7 | -1.825e+01 | 0.0000 | 1165.0 | 12.53% | 3483.7 | 10.70% | motif file (matrix) | svg |
| 64 | C T A G C T A G A G T C T C A G A C T G A C G T A C G T C T G A | MYB(HTH)/ERMYB-Myb-ChIPSeq(GSE22095)/Homer | 1e-7 | -1.779e+01 | 0.0000 | 4172.0 | 44.87% | 13693.3 | 42.04% | motif file (matrix) | svg |
| 65 | C T A G T A C G A G T C C G T A A G T C A C G T A G T C T C G A C G T A T A C G | Nkx2.1(Homeobox)/LungAC-Nkx2.1-ChIP-Seq(GSE43252)/Homer | 1e-7 | -1.756e+01 | 0.0000 | 5833.0 | 62.73% | 19531.5 | 59.97% | motif file (matrix) | svg |
| 66 | C G T A T A C G T C G A A C T G A C T G C G T A C G T A T A C G A G C T T A C G | PU.1(ETS)/ThioMac-PU.1-ChIP-Seq(GSE21512)/Homer | 1e-7 | -1.711e+01 | 0.0000 | 1307.0 | 14.06% | 3970.3 | 12.19% | motif file (matrix) | svg |
| 67 | T G A C C T G A C T A G T C G A C T G A A T G C C G T A A C T G G C A T G T A C G C A T A T C G G C A T A G C T G A T C | PR(NR)/T47D-PR-ChIP-Seq(GSE31130)/Homer | 1e-7 | -1.669e+01 | 0.0000 | 5250.0 | 56.46% | 17497.7 | 53.72% | motif file (matrix) | svg |
| 68 | C G T A A C G T A C G T A C G T A C G T A G T C A G T C C T G A A G C T A G C T | NFAT(RHD)/Jurkat-NFATC1-ChIP-Seq(Jolma\_et\_al.)/Homer | 1e-7 | -1.658e+01 | 0.0000 | 2950.0 | 31.73% | 9514.2 | 29.21% | motif file (matrix) | svg |
| 69 | T C G A T C G A T A C G G A T C G T C A G T A C C G A T A G C T G T C A T G C A | Nkx3.1(Homeobox)/LNCaP-Nkx3.1-ChIP-Seq(GSE28264)/Homer | 1e-7 | -1.616e+01 | 0.0000 | 5339.0 | 57.42% | 17827.9 | 54.74% | motif file (matrix) | svg |
| 70 | A T G C G A C T A C T G C A G T G A T C A C G T T A C G T A C G | Smad2(MAD)/ES-SMAD2-ChIP-Seq(GSE29422)/Homer | 1e-6 | -1.596e+01 | 0.0000 | 3430.0 | 36.89% | 11180.0 | 34.32% | motif file (matrix) | svg |
| 71 | C T A G T C G A C G A T C T A G G C A T C A G T C T A G G A T C C G T A G T C A | CEBP:AP1(bZIP)/ThioMac-CEBPb-ChIP-Seq(GSE21512)/Homer | 1e-6 | -1.452e+01 | 0.0000 | 2479.0 | 26.66% | 7966.0 | 24.46% | motif file (matrix) | svg |
| 72 | T A C G T C A G A G C T A T G C C G T A A G T C T C A G A C G T A C T G T C G A | USF1(bHLH)/GM12878-Usf1-ChIP-Seq(GSE32465)/Homer | 1e-6 | -1.431e+01 | 0.0000 | 872.0 | 9.38% | 2598.1 | 7.98% | motif file (matrix) | svg |
| 73 | A G C T A G C T C A T G C T G A G T A C A G T C A G C T A G C T C A G T C T A G | RARa(NR)/K562-RARa-ChIP-Seq(Encode)/Homer | 1e-6 | -1.423e+01 | 0.0000 | 4974.0 | 53.50% | 16606.3 | 50.98% | motif file (matrix) | svg |
| 74 | C A T G G A C T T A C G G T C A G T A C G A T C G A C T A G C T A T C G T C G A T A C G T A G C | ERRg(NR)/Kidney-ESRRG-ChIP-Seq(GSE104905)/Homer | 1e-6 | -1.403e+01 | 0.0000 | 1501.0 | 16.14% | 4679.3 | 14.37% | motif file (matrix) | svg |
| 75 | T C G A C T A G A G T C A G T C C G T A C G T A A C G T T A G C T C A G T A C G | NFY(CCAAT)/Promoter/Homer | 1e-5 | -1.373e+01 | 0.0000 | 1780.0 | 19.14% | 5621.2 | 17.26% | motif file (matrix) | svg |
| 76 | T C G A T G A C G C A T A G C T C A G T G A T C G C T A G A T C G A C T A C G T G C A T A G T C | PRDM1(Zf)/Hela-PRDM1-ChIP-Seq(GSE31477)/Homer | 1e-5 | -1.352e+01 | 0.0000 | 1779.0 | 19.13% | 5624.0 | 17.27% | motif file (matrix) | svg |
| 77 | C A G T A C T G T C A G T G C A G C T A A T G C T C G A A T C G G T C A T G C A | ZNF189(Zf)/HEK293-ZNF189.GFP-ChIP-Seq(GSE58341)/Homer | 1e-5 | -1.329e+01 | 0.0000 | 2172.0 | 23.36% | 6957.3 | 21.36% | motif file (matrix) | svg |
| 78 | A T G C G A C T A G C T C T A G C G T A C T A G C G A T C T A G A T C G G A T C | Nkx2.2(Homeobox)/NPC-Nkx2.2-ChIP-Seq(GSE61673)/Homer | 1e-5 | -1.326e+01 | 0.0000 | 4144.0 | 44.57% | 13740.0 | 42.18% | motif file (matrix) | svg |
| 79 | C G A T T G C A G T A C C G T A A G T C C T A G G A C T C A T G | NPAS(bHLH)/Liver-NPAS-ChIP-Seq(GSE39860)/Homer | 1e-5 | -1.306e+01 | 0.0000 | 3748.0 | 40.31% | 12371.7 | 37.98% | motif file (matrix) | svg |
| 80 | A G C T G A C T C T A G C G T A C A T G C G A T C T A G A T C G G A C T C A G T | Bapx1(Homeobox)/VertebralCol-Bapx1-ChIP-Seq(GSE36672)/Homer | 1e-5 | -1.216e+01 | 0.0000 | 4771.0 | 51.31% | 15967.8 | 49.02% | motif file (matrix) | svg |
| 81 | T C A G T C A G G C T A C G T A T A C G G A C T T C A G T C G A C T G A C G T A T A C G G A C T | IRF8(IRF)/BMDM-IRF8-ChIP-Seq(GSE77884)/Homer | 1e-5 | -1.168e+01 | 0.0000 | 1057.0 | 11.37% | 3258.0 | 10.00% | motif file (matrix) | svg |
| 82 | C T G A C T A G A T C G G C A T A C T G G T A C A T G C C G T A A C T G G C T A A G T C C G T A | Tbox:Smad(T-box,MAD)/ESCd5-Smad2\_3-ChIP-Seq(GSE29422)/Homer | 1e-5 | -1.167e+01 | 0.0000 | 526.0 | 5.66% | 1525.6 | 4.68% | motif file (matrix) | svg |
| 83 | A T G C C G T A A C T G C G T A A C G T G C T A T C G A A G C T C G A T C G T A A C G T A G T C C G A T A C T G G A T C | GATA(Zf),IR4/iTreg-Gata3-ChIP-Seq(GSE20898)/Homer | 1e-5 | -1.156e+01 | 0.0001 | 296.0 | 3.18% | 802.1 | 2.46% | motif file (matrix) | svg |
| 84 | T G C A C T G A A T G C G T C A A C T G A C T G C G T A C G T A C T A G A G C T | Ets1-distal(ETS)/CD4+-PolII-ChIP-Seq(Barski\_et\_al.)/Homer | 1e-5 | -1.152e+01 | 0.0001 | 891.0 | 9.58% | 2713.8 | 8.33% | motif file (matrix) | svg |
| 85 | T C G A T G C A A C T G G T C A A G T C A G T C A G T C A G T C A G C T T G A C | LRF(Zf)/Erythroblasts-ZBTB7A-ChIP-Seq(GSE74977)/Homer | 1e-4 | -1.147e+01 | 0.0001 | 1578.0 | 16.97% | 5001.2 | 15.35% | motif file (matrix) | svg |
| 86 | G A C T C T A G C T A G A G T C T G C A A C T G A C G T A C G T C T A G T C A G | AMYB(HTH)/Testes-AMYB-ChIP-Seq(GSE44588)/Homer | 1e-4 | -1.097e+01 | 0.0001 | 3738.0 | 40.20% | 12411.2 | 38.10% | motif file (matrix) | svg |
| 87 | C A T G G T A C C G T A A G T C C T A G A C G T A C T G G T A C A G T C A G C T | bHLHE40(bHLH)/HepG2-BHLHE40-ChIP-Seq(GSE31477)/Homer | 1e-4 | -1.096e+01 | 0.0001 | 526.0 | 5.66% | 1536.3 | 4.72% | motif file (matrix) | svg |
| 88 | C T A G C T A G C G T A C G T A T A C G C G A T C T A G C T G A C T G A C G T A T A C G G A C T | PU.1:IRF8(ETS:IRF)/pDC-Irf8-ChIP-Seq(GSE66899)/Homer | 1e-4 | -1.085e+01 | 0.0001 | 624.0 | 6.71% | 1854.7 | 5.69% | motif file (matrix) | svg |
| 89 | A T G C T A G C A G C T A G C T T G A C G A C T T C A G T A C G G T C A C T G A A T C G T A G C G A C T C A G T A G T C A G C T T C G A A T C G T G C A T G C A | HRE(HSF)/HepG2-HSF1-ChIP-Seq(GSE31477)/Homer | 1e-4 | -1.061e+01 | 0.0001 | 421.0 | 4.53% | 1206.7 | 3.70% | motif file (matrix) | svg |
| 90 | C A T G A G T C C T G A T G A C A T C G G A C T G T C A A G T C T A G C G A T C | HIF2a(bHLH)/785\_O-HIF2a-ChIP-Seq(GSE34871)/Homer | 1e-4 | -1.031e+01 | 0.0002 | 633.0 | 6.81% | 1893.3 | 5.81% | motif file (matrix) | svg |
| 91 | C T G A C T G A T A G C G A T C G C T A G T A C A C G T G A T C T G C A C G T A | Nkx2.5(Homeobox)/HL1-Nkx2.5.biotin-ChIP-Seq(GSE21529)/Homer | 1e-4 | -1.023e+01 | 0.0002 | 4805.0 | 51.68% | 16160.8 | 49.62% | motif file (matrix) | svg |
| 92 | A C T G G A T C G A C T A C T G A C G T C A T G A C T G A C G T A G C T C G A T | RUNX-AML(Runt)/CD4+-PolII-ChIP-Seq(Barski\_et\_al.)/Homer | 1e-4 | -1.019e+01 | 0.0002 | 1797.0 | 19.33% | 5777.7 | 17.74% | motif file (matrix) | svg |
| 93 | T C G A T A G C T G A C C T G A A G T C A C T G G A C T C A T G | c-Myc(bHLH)/LNCAP-cMyc-ChIP-Seq(Unpublished)/Homer | 1e-4 | -1.014e+01 | 0.0002 | 675.0 | 7.26% | 2033.9 | 6.24% | motif file (matrix) | svg |
| 94 | T G C A A G C T A C G T C T A G G A T C C T A G G A T C G T C A C T G A A G T C | CEBP(bZIP)/ThioMac-CEBPb-ChIP-Seq(GSE21512)/Homer | 1e-4 | -9.984e+00 | 0.0002 | 2189.0 | 23.54% | 7116.2 | 21.85% | motif file (matrix) | svg |
| 95 | G A C T A G C T G T A C G A C T C T G A A C T G G T C A C T G A A T G C T A C G G A C T A C G T A G T C G A C T C T G A | HRE(HSF)/Striatum-HSF1-ChIP-Seq(GSE38000)/Homer | 1e-4 | -9.671e+00 | 0.0003 | 733.0 | 7.88% | 2232.1 | 6.85% | motif file (matrix) | svg |
| 96 | G C T A A G T C T A C G T G C A A T C G T C A G G C T A T C G A T C A G A G C T | ELF5(ETS)/T47D-ELF5-ChIP-Seq(GSE30407)/Homer | 1e-4 | -9.392e+00 | 0.0004 | 1956.0 | 21.04% | 6342.2 | 19.47% | motif file (matrix) | svg |
| 97 | C G T A C T G A A C T G A G C T A G T C G A T C G A T C G C A T C T G A C T A G C T A G T A C G T C G A T G C A G C A T | EBF2(EBF)/BrownAdipose-EBF2-ChIP-Seq(GSE97114)/Homer | 1e-4 | -9.333e+00 | 0.0004 | 1752.0 | 18.84% | 5651.9 | 17.35% | motif file (matrix) | svg |
| 98 | C T G A T G C A T A G C T G A C T A C G T C A G C T G A G C T A T C A G G A C T | ELF1(ETS)/Jurkat-ELF1-ChIP-Seq(SRA014231)/Homer | 1e-4 | -9.275e+00 | 0.0004 | 840.0 | 9.03% | 2593.5 | 7.96% | motif file (matrix) | svg |
| 99 | A T G C A G T C A G C T A G C T A C G T A T C G C G T A C G A T T A G C G A C T | LEF1(HMG)/H1-LEF1-ChIP-Seq(GSE64758)/Homer | 1e-3 | -8.544e+00 | 0.0009 | 2174.0 | 23.38% | 7116.0 | 21.85% | motif file (matrix) | svg |
| 100 | C G A T C T A G T C A G C A G T C G T A A G T C G C T A A C G T G A C T A T G C A G T C G C T A | PRDM10(Zf)/HEK293-PRDM10.eGFP-ChIP-Seq(Encode)/Homer | 1e-3 | -8.404e+00 | 0.0010 | 1322.0 | 14.22% | 4226.8 | 12.98% | motif file (matrix) | svg |
| 101 | G T C A C T G A T C A G G A T C T G C A T G C A A C G T T C A G C G T A C G T A C G T A G C T A | Hoxd12(Homeobox)/ChickenMSG-Hoxd12.Flag-ChIP-Seq(GSE86088)/Homer | 1e-3 | -8.278e+00 | 0.0011 | 5300.0 | 57.00% | 17981.5 | 55.21% | motif file (matrix) | svg |
| 102 | C T A G C A T G T G C A A C G T A G T C C G T A C A T G T C A G A C G T A C G T G C T A A G T C | Six1(Homeobox)/Myoblast-Six1-ChIP-Chip(GSE20150)/Homer | 1e-3 | -8.268e+00 | 0.0011 | 739.0 | 7.95% | 2283.0 | 7.01% | motif file (matrix) | svg |
| 103 | G A T C C T G A A G T C C G A T C G A T G A T C A G T C A C T G A T C G A G C T | Elk4(ETS)/Hela-Elk4-ChIP-Seq(GSE31477)/Homer | 1e-3 | -8.261e+00 | 0.0011 | 948.0 | 10.20% | 2976.2 | 9.14% | motif file (matrix) | svg |
| 104 | C T G A A T G C G C T A C G A T A T G C C G T A C G T A C G T A C T A G T A C G | Tcf3(HMG)/mES-Tcf3-ChIP-Seq(GSE11724)/Homer | 1e-3 | -8.208e+00 | 0.0012 | 837.0 | 9.00% | 2608.8 | 8.01% | motif file (matrix) | svg |
| 105 | C T G A T C A G G C T A A G C T A G T C G A C T C T G A C T A G T G C A C T G A A G T C G T A C G A T C A C T G T C G A | ZBTB12(Zf)/HEK293-ZBTB12.GFP-ChIP-Seq(GSE58341)/Homer | 1e-3 | -8.158e+00 | 0.0012 | 949.0 | 10.21% | 2982.2 | 9.16% | motif file (matrix) | svg |
| 106 | C T A G T A C G G A C T T G C A T G C A C G A T T A C G C T G A T C G A C T G A | Hoxa10(Homeobox)/ChickenMSG-Hoxa10.Flag-ChIP-Seq(GSE86088)/Homer | 1e-3 | -8.072e+00 | 0.0013 | 2003.0 | 21.54% | 6548.8 | 20.11% | motif file (matrix) | svg |
| 107 | G A T C T C G A A G T C C G A T C G A T A G T C A T G C A C T G A T C G G A C T | Elk1(ETS)/Hela-Elk1-ChIP-Seq(GSE31477)/Homer | 1e-3 | -7.976e+00 | 0.0014 | 920.0 | 9.89% | 2890.0 | 8.87% | motif file (matrix) | svg |
| 108 | C T A G C A T G C A T G T A C G A G T C G C A T A G C T C T A G A C G T A G T C G A C T A C T G A C T G A C T G T C G A | Zfp809(Zf)/ES-Zfp809-ChIP-Seq(GSE70799)/Homer | 1e-3 | -7.883e+00 | 0.0015 | 250.0 | 2.69% | 703.8 | 2.16% | motif file (matrix) | svg |
| 109 | C T A G G C A T G A T C C G T A A G T C T C A G G A C T C T A G | CLOCK(bHLH)/Liver-Clock-ChIP-Seq(GSE39860)/Homer | 1e-3 | -7.857e+00 | 0.0016 | 1124.0 | 12.09% | 3576.8 | 10.98% | motif file (matrix) | svg |
| 110 | A G T C T G C A T C G A C T G A A C T G C A T G A C G T A T G C G T C A T A C G | Erra(NR)/HepG2-Erra-ChIP-Seq(GSE31477)/Homer | 1e-3 | -7.812e+00 | 0.0016 | 3429.0 | 36.88% | 11468.1 | 35.21% | motif file (matrix) | svg |
| 111 | T A C G C T G A T C G A C G A T C T A G C T A G T C G A C T G A T C G A T C G A C G T A T C G A G C A T C A T G C G T A T A C G G C A T T G A C C G T A A G C T | NFAT:AP1(RHD,bZIP)/Jurkat-NFATC1-ChIP-Seq(Jolma\_et\_al.)/Homer | 1e-3 | -7.596e+00 | 0.0020 | 632.0 | 6.80% | 1944.3 | 5.97% | motif file (matrix) | svg |
| 112 | C T A G C A G T T G A C C G T A G A T C T C A G G A C T C A T G | BMAL1(bHLH)/Liver-Bmal1-ChIP-Seq(GSE39860)/Homer | 1e-3 | -7.435e+00 | 0.0023 | 4087.0 | 43.96% | 13773.0 | 42.29% | motif file (matrix) | svg |
| 113 | G C T A T A G C A G C T A T C G G T C A C G T A G C T A A T G C G A T C C T G A | IRF4(IRF)/GM12878-IRF4-ChIP-Seq(GSE32465)/Homer | 1e-3 | -7.362e+00 | 0.0025 | 1279.0 | 13.76% | 4113.3 | 12.63% | motif file (matrix) | svg |
| 114 | C A T G A G C T T A C G G T C A G T A C T A G C A G C T G A C T A T C G T C G A | Esrrb(NR)/mES-Esrrb-ChIP-Seq(GSE11431)/Homer | 1e-3 | -7.216e+00 | 0.0028 | 1184.0 | 12.73% | 3797.7 | 11.66% | motif file (matrix) | svg |
| 115 | T A C G A C T G A G C T G T A C C G T A T C G A C T G A A C T G C A T G A C G T A G T C C G T A | COUP-TFII(NR)/K562-NR2F1-ChIP-Seq(Encode)/Homer | 1e-3 | -7.152e+00 | 0.0030 | 2138.0 | 22.99% | 7045.6 | 21.63% | motif file (matrix) | svg |
| 116 | T G A C G C T A T C G A T G C A A G T C A G T C C G T A A G T C C G T A C T G A G C T A G T A C | RUNX2(Runt)/PCa-RUNX2-ChIP-Seq(GSE33889)/Homer | 1e-3 | -7.123e+00 | 0.0031 | 2100.0 | 22.59% | 6916.3 | 21.23% | motif file (matrix) | svg |
| 117 | C A T G A C G T A G T C G A T C G A T C G A T C G C T A C T A G C T A G C T A G T C A G T C G A | EBF1(EBF)/Near-E2A-ChIP-Seq(GSE21512)/Homer | 1e-3 | -7.049e+00 | 0.0033 | 1618.0 | 17.40% | 5273.9 | 16.19% | motif file (matrix) | svg |
| 118 | A C T G G A T C C T G A A T C G A G T C T A G C C T G A C G T A T A C G A G T C C T A G C A G T T C A G T C G A T G A C G A T C | PAX5(Paired,Homeobox)/GM12878-PAX5-ChIP-Seq(GSE32465)/Homer | 1e-3 | -7.027e+00 | 0.0033 | 684.0 | 7.36% | 2129.6 | 6.54% | motif file (matrix) | svg |
| 119 | T A C G A T C G T A G C G A T C A C T G A C G T A G T C A C G T C T A G A T C G | Smad4(MAD)/ESC-SMAD4-ChIP-Seq(GSE29422)/Homer | 1e-3 | -6.994e+00 | 0.0034 | 3415.0 | 36.73% | 11457.3 | 35.18% | motif file (matrix) | svg |
| 120 | T G C A T C G A T A G C G T A C T C A G C T A G G T C A G C T A T C A G G A C T | ETS(ETS)/Promoter/Homer | 1e-3 | -6.950e+00 | 0.0035 | 553.0 | 5.95% | 1698.6 | 5.21% | motif file (matrix) | svg |
| 121 | T C A G T A C G T A G C A C G T A C T G C G A T A G T C C G T A T A C G A G T C | Meis1(Homeobox)/MastCells-Meis1-ChIP-Seq(GSE48085)/Homer | 1e-2 | -6.907e+00 | 0.0036 | 3844.0 | 41.34% | 12952.5 | 39.77% | motif file (matrix) | svg |
| 122 | C T G A T C A G G T A C T G C A A G T C C G T A A G T C A C T G A C G T A C T G | MNT(bHLH)/HepG2-MNT-ChIP-Seq(Encode)/Homer | 1e-2 | -6.903e+00 | 0.0036 | 2548.0 | 27.40% | 8464.5 | 25.99% | motif file (matrix) | svg |
| 123 | A G T C G A C T A C T G G A T C G T A C C G T A T G A C A G T C C G A T A G C T A C G T A C G T C T A G G A C T C T G A | ZNF7(Zf)/HepG2-ZNF7.Flag-ChIP-Seq(Encode)/Homer | 1e-2 | -6.756e+00 | 0.0042 | 1770.0 | 19.04% | 5802.6 | 17.82% | motif file (matrix) | svg |
| 124 | C G T A C T G A C G T A C T A G T C G A C T A G A C T G C G T A C G T A T A C G A G C T A T C G | SpiB(ETS)/OCILY3-SPIB-ChIP-Seq(GSE56857)/Homer | 1e-2 | -6.731e+00 | 0.0042 | 655.0 | 7.04% | 2040.2 | 6.26% | motif file (matrix) | svg |
| 125 | G C T A C G A T C A T G A T G C A G T C A G T C G A C T T A C G T C G A C T A G A C T G T A G C | AP-2alpha(AP2)/Hela-AP2alpha-ChIP-Seq(GSE31477)/Homer | 1e-2 | -6.655e+00 | 0.0045 | 880.0 | 9.46% | 2792.9 | 8.57% | motif file (matrix) | svg |
| 126 | A G C T A G C T T A G C A T C G A G T C A C T G A T G C A T C G T C G A C T G A T C G A C T G A | E2F(E2F)/Hela-CellCycle-Expression/Homer | 1e-2 | -6.468e+00 | 0.0054 | 42.0 | 0.45% | 89.4 | 0.27% | motif file (matrix) | svg |
| 127 | G C A T C T A G G T A C A G T C C G A T A C T G C T A G C T A G G T A C G C T A | ZNF416(Zf)/HEK293-ZNF416.GFP-ChIP-Seq(GSE58341)/Homer | 1e-2 | -6.462e+00 | 0.0054 | 2687.0 | 28.90% | 8964.0 | 27.52% | motif file (matrix) | svg |
| 128 | T A G C G T A C C G T A C T A G A C T G T G C A C G T A A T G C C G T A A T C G | AR-halfsite(NR)/LNCaP-AR-ChIP-Seq(GSE27824)/Homer | 1e-2 | -6.434e+00 | 0.0055 | 6917.0 | 74.39% | 23789.3 | 73.04% | motif file (matrix) | svg |
| 129 | C G T A A C T G G T C A A C G T A T C G C A G T C T A G T C A G C G T A A C T G C G T A A C G T C G T A C T G A T A C G | GATA3(Zf),DR4/iTreg-Gata3-ChIP-Seq(GSE20898)/Homer | 1e-2 | -6.408e+00 | 0.0056 | 332.0 | 3.57% | 987.1 | 3.03% | motif file (matrix) | svg |
| 130 | A C T G A G C T A G T C G T C A A G C T T C A G A T G C G A T C G C A T A T C G T C G A T A G C C G A T C A T G T A G C | Pax8(Paired,Homeobox)/Thyroid-Pax8-ChIP-Seq(GSE26938)/Homer | 1e-2 | -6.310e+00 | 0.0062 | 594.0 | 6.39% | 1848.5 | 5.68% | motif file (matrix) | svg |
| 131 | T C A G G C T A T C A G C A G T T G A C G T C A A G T C A T C G T G C A G T A C C A G T G A T C | Npas4(bHLH)/Neuron-Npas4-ChIP-Seq(GSE127793)/Homer | 1e-2 | -6.140e+00 | 0.0072 | 1383.0 | 14.87% | 4507.3 | 13.84% | motif file (matrix) | svg |
| 132 | C G A T C T G A C G T A A G C T A G T C T G C A C T G A A C G T A G C T G C T A | Pax7(Paired,Homeobox)/Myoblast-Pax7-ChIP-Seq(GSE25064)/Homer | 1e-2 | -6.057e+00 | 0.0078 | 401.0 | 4.31% | 1218.9 | 3.74% | motif file (matrix) | svg |
| 133 | A T G C G A T C G A C T A G C T C G A T C G A T G T C A C G A T T C G A A T C G T A G C T A G C | TATA-Box(TBP)/Promoter/Homer | 1e-2 | -5.960e+00 | 0.0085 | 3967.0 | 42.67% | 13429.2 | 41.23% | motif file (matrix) | svg |
| 134 | C T A G C A T G A C G T A G T C G C T A A G C T A G T C A G C T T C A G C T G A A C T G C A T G G C A T A T G C C G T A | THRa(NR)/C17.2-THRa-ChIP-Seq(GSE38347)/Homer | 1e-2 | -5.936e+00 | 0.0087 | 606.0 | 6.52% | 1897.6 | 5.83% | motif file (matrix) | svg |
| 135 | C T A G T A C G G A T C G T C A T C G A A C G T T C A G C G T A C G T A C G T A | Hoxd10(Homeobox)/ChickenMSG-Hoxd10.Flag-ChIP-Seq(GSE86088)/Homer | 1e-2 | -5.859e+00 | 0.0093 | 3477.0 | 37.40% | 11729.5 | 36.01% | motif file (matrix) | svg |
| 136 | G A C T C T A G C T A G C T A G A C T G T C G A C T G A C T A G C T A G C T A G G T A C G T C A | ZNF467(Zf)/HEK293-ZNF467.GFP-ChIP-Seq(GSE58341)/Homer | 1e-2 | -5.689e+00 | 0.0109 | 1398.0 | 15.04% | 4575.8 | 14.05% | motif file (matrix) | svg |
| 137 | T C A G A G C T A T G C C G T A A G C T T C A G C A G T A C T G C T G A A G T C | MITF(bHLH)/MastCells-MITF-ChIP-Seq(GSE48085)/Homer | 1e-2 | -5.664e+00 | 0.0111 | 2035.0 | 21.89% | 6755.1 | 20.74% | motif file (matrix) | svg |
| 138 | A G T C C T G A A T C G A G C T A G C T G A C T A G T C G C T A A C G T C G A T G C A T C G A T A T C G C G T A T A G C G C A T A T G C C G T A | bZIP:IRF(bZIP,IRF)/Th17-BatF-ChIP-Seq(GSE39756)/Homer | 1e-2 | -5.658e+00 | 0.0111 | 1681.0 | 18.08% | 5542.5 | 17.02% | motif file (matrix) | svg |
| 139 | T C A G C A T G C A T G A C T G A C T G A G C T A C T G A C G T A C T G C A G T A T G C A G T C | KLF10(Zf)/HEK293-KLF10.GFP-ChIP-Seq(GSE58341)/Homer | 1e-2 | -5.487e+00 | 0.0131 | 1257.0 | 13.52% | 4103.6 | 12.60% | motif file (matrix) | svg |
| 140 | A T C G A G T C A G T C G A C T A T G C C T G A C T A G A C T G T A C G G T A C C T G A C G A T | AP-2gamma(AP2)/MCF7-TFAP2C-ChIP-Seq(GSE21234)/Homer | 1e-2 | -5.372e+00 | 0.0146 | 1226.0 | 13.19% | 4002.3 | 12.29% | motif file (matrix) | svg |
| 141 | C T G A T A C G G C A T A G C T A G C T A G T C T C G A A C T G C A G T A G C T A G C T G A T C | IRF3(IRF)/BMDM-Irf3-ChIP-Seq(GSE67343)/Homer | 1e-2 | -5.206e+00 | 0.0171 | 1160.0 | 12.48% | 3784.9 | 11.62% | motif file (matrix) | svg |
| 142 | T A C G T A C G G T A C A T C G T A C G T A C G G T C A C T G A C G T A G A C T | E2F4(E2F)/K562-E2F4-ChIP-Seq(GSE31477)/Homer | 1e-2 | -5.057e+00 | 0.0197 | 312.0 | 3.36% | 947.2 | 2.91% | motif file (matrix) | svg |
| 143 | C A G T G A C T A G T C G C T A C T G A G T C A C G T A A G C T G T C A C T G A C T G A C T G A A C T G A G C T G A T C | MYNN(Zf)/HEK293-MYNN.eGFP-ChIP-Seq(Encode)/Homer | 1e-2 | -4.997e+00 | 0.0208 | 964.0 | 10.37% | 3127.4 | 9.60% | motif file (matrix) | svg |
| 144 | T C A G A C G T A G T C T C G A A G T C T C A G G C A T C T A G C T A G A G C T | Usf2(bHLH)/C2C12-Usf2-ChIP-Seq(GSE36030)/Homer | 1e-2 | -4.977e+00 | 0.0211 | 665.0 | 7.15% | 2120.7 | 6.51% | motif file (matrix) | svg |
| 145 | G T A C G C T A T C A G C T G A C T A G C A T G A G C T G A T C T G C A T C G A C T G A A C T G C A G T A G T C G A T C G C T A | HNF4a(NR),DR1/HepG2-HNF4a-ChIP-Seq(GSE25021)/Homer | 1e-2 | -4.835e+00 | 0.0241 | 897.0 | 9.65% | 2907.0 | 8.93% | motif file (matrix) | svg |
| 146 | T A C G T C A G G A T C G T A C T C G A G A C T G C T A G C T A G C T A C G T A G A T C G T C A | CDX4(Homeobox)/ZebrafishEmbryos-Cdx4.Myc-ChIP-Seq(GSE48254)/Homer | 1e-2 | -4.697e+00 | 0.0275 | 3053.0 | 32.84% | 10321.9 | 31.69% | motif file (matrix) | svg |
| 147 | T A C G C T G A C A T G G A T C G T A C G C A T T C A G T A C G A G C T G T C A G A T C G C A T T A C G C G T A C T A G G A T C G A T C C G A T A C T G T C A G | ZNF322(Zf)/HEK293-ZNF322.GFP-ChIP-Seq(GSE58341)/Homer | 1e-2 | -4.679e+00 | 0.0278 | 455.0 | 4.89% | 1427.4 | 4.38% | motif file (matrix) | svg |
| 148 | A G T C G A C T A G C T C G A T A T C G G C T A C G A T A T C G C G A T A C T G T A C G A C G T | Tcf7(HMG)/GM12878-TCF7-ChIP-Seq(Encode)/Homer | 1e-2 | -4.643e+00 | 0.0286 | 1013.0 | 10.89% | 3306.0 | 10.15% | motif file (matrix) | svg |
| 149 | T A C G T A C G G T A C A T C G A C T G T A C G T C G A C T G A T C G A A T C G | E2F6(E2F)/Hela-E2F6-ChIP-Seq(GSE31477)/Homer | 1e-2 | -4.619e+00 | 0.0291 | 493.0 | 5.30% | 1555.1 | 4.77% | motif file (matrix) | svg |
